# HMGB1-dependent and -independent autophagy

**DOI:** 10.4161/auto.32184

**Published:** 2014-08-13

**Authors:** Xiaofang Sun, Daolin Tang

**Affiliations:** 1 Key Laboratory for Major Obstetric Diseases of Guangdong Province; Key Laboratory of Reproduction and Genetics of Guangdong Higher Education Institutes; The Third Affiliated Hospital of Guangzhou Medical University; Guangzhou, Guangdong China; 2 Department of Surgery; University of Pittsburgh Cancer Institute; University of Pittsburgh; Pittsburgh, PA USA

**Keywords:** autophagy, HMGB1, knockin, knockout, phenotype

## Abstract

HMGB1 (high mobility group box 1) is a multifunctional, ubiquitous protein located inside and outside cells that plays a critical role in various physiological and pathological processes including cell development, differentiation, inflammation, immunity, metastasis, metabolism, and death. Increasing evidence demonstrates that HMGB1-dependent autophagy promotes chemotherapy resistance, sustains tumor metabolism requirements and T cell survival, prevents polyglutamine aggregates and excitotoxicity, and protects against endotoxemia, bacterial infection, and ischemia-reperfusion injury in vitro or in vivo. In contrast, HMGB1 may not be required for autophagy in some organs such as the liver and heart. Understanding HMGB1-dependent and -independent autophagy in more detail will provide insight into the integrated stress response and guide HMGB1-based therapeutic intervention.

HMGB1 is an evolutionarily ancient protein that possibly originated more than 525 million years ago before the protostomes and deuterostomes split. It was first identified in 1973 by Ernest Johns and coworkers as one of a group of nonhistone, chromatin-associated proteins with 2 DNA-binding HMG-box domains (A and B box) and an acidic C-terminal tail.^1^ HMGB1 is normally located in the nucleus, acting as a DNA chaperone involved in the regulation of a number of DNA-associated processes such as replication, transcription, recombination, and repair. In addition to its nuclear function, HMGB1 can act as a stress sensor and translocate from the nucleus to the cytoplasm and then be released into the extracellular space during various stress conditions. Autophagy is generally a programmed cell survival process and lysosome-mediated pathway involving the degradation of cellular components (e.g., long-lived proteins and damaged organelles) and invading pathogens in a selective or nonselective manner.^2^^-^^4^ The dynamic process of autophagy is primarily controlled by the autophagy-related (ATG) protein family, and it shares regulators from other trafficking pathways and cell death.^4^^,^^5^ In the past few years, increasing evidence supports the existence of ATG pathway (e.g., ATG5, ATG7, and BECN1)-independent autophagy, making the autophagy machinery as well as autophagy monitoring extremely complicated.^6^^-^^8^ Indeed, HMGB1 has a context-dependent role in the regulation of autophagy and stress.^9^ Here, we outline the exciting new advances in our knowledge of HMGB1-dependent and -independent autophagy and discuss how these advances are driving the understanding of the integrated stress response.

## HMGB1-Dependent Autophagy

HMGB1 participates in the autophagy process at several levels (Fig. 1A). First, HMGB1 translocates to the cytoplasm following several autophagic stimuli (e.g., hydrogen peroxide, rapamycin, and starvation), which in turn promotes autophagy through direct interaction with BECN1 to dissociate it from BCL2 in immortalized mouse embryonic fibroblasts and cancer cells.^10^ Meanwhile, HMGB1 C23S and C45S mutants lose their ability to mediate autophagy, as they are unable to bind BECN1 and therefore cannot disrupt BCL2-BECN1 interactions.^10^ In addition, the HMGB1-BECN1 complex seems to be tightly controlled at the transcriptional, post-transcriptional, post-translational, and protein-protein interaction level. For example, ULK1 (unc-51 like autophagy activating kinase 1),^11^ MAPK (mitogen-activated protein kinase),^10^ and NACC1 (nucleus accumbens associated 1, BEN and BTB [POZ] domain containing)^12^ positively regulate HMGB1-mediated autophagy, whereas TP53,^13^ SNCA/α-synuclein,^14^ IFI30/gamma-interferon-inducible lysosomal thiol reductase,^15^ MIR34A,^16^ and MIR22^17^ negatively regulate HMGB1-mediated autophagy. Second, HMGB1 regulates the expression of HSPB1 (heat shock 27 kDa protein 1) in immortalized mouse embryonic fibroblasts and cancer cells.^18^ As a cytoskeleton regulator, HSPB1 is important for dynamic intracellular trafficking during autophagy and mitophagy. Thus, inhibition of the HMGB1-HSPB1 pathway impairs mitophagy and elimination of damaged mitochondria in response to mitochondrial electron-transport-chain inhibitors.^18^ Third, extracellular reduced HMGB1 induces autophagy and tumor growth through AGER/RAGE (advanced glycosylation end product-specific receptor), whereas oxidized HMGB1 induces apoptosis in cancer cells.^19^ HMGB1 released from cancer cells induces autophagy in the muscle, which sustains anaerobic energy production (namely the Warburg effect) during tumor growth in vitro and in vivo.^20^ These findings suggest that HMGB1 is an important mediator of systemic autophagic syndrome.

**Figure:**
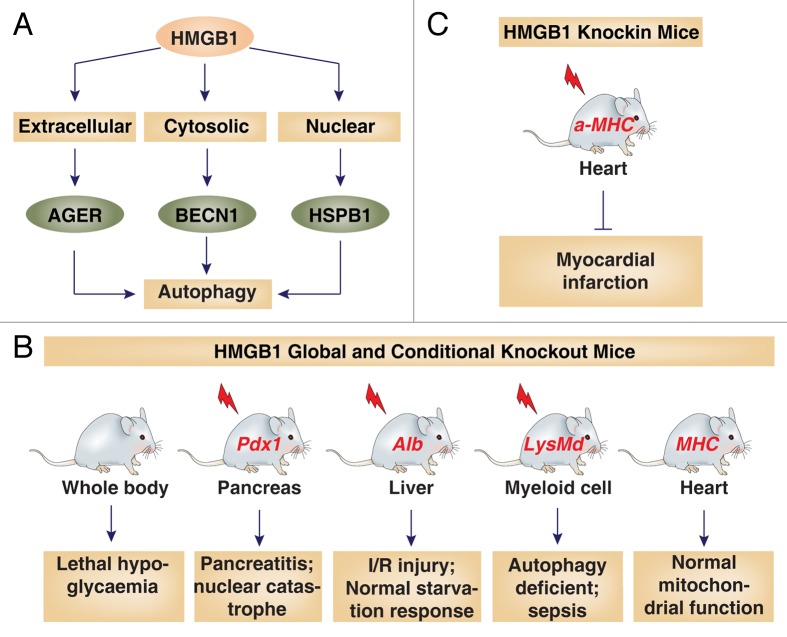
**Figure 1.** HMGB1 is involved in autophagy and other stress responses. (**A**) HMGB1 plays important nuclear, cytosolic, and extracellular roles in the regulation of autophagy. (**B and C**) Various phenotypes of HMGB1 knockout (**B**) and knockin (**C**) mice with or without stress (indicated by lightning bolt).

## HMGB1-Independent Autophagy

HMGB1 global knockout mice die shortly after birth due to the downregulation of glucocorticoid receptor and subsequent hypoglycemia, suggesting a critical role for HMGB1 in sustaining life.^21^ We and others recently generated transgenic mice with conditional knockout (Fig. 1B) or knockin (Fig. 1C) of HMGB1 within the pancreas,^22^ liver,^23^^,^^24^ heart,^24^^,^^25^ and myeloid cells^26^ through a different strategy. All these mice were viable and had no significant defects such as glucose and energy metabolism defects under unstressed growth conditions. However, these mice have various, even opposite, phenotypes in response to different stressors. For example, knockout of HMGB1 in the pancreas (n = 18–25 mice per group), liver (n = 6 mice per group), and myeloid cells (n = 6–9 mice per group) make mice more sensitive to sterile inflammation (e.g., pancreatitis^22^ and liver ischemic reperfusion^23^) and infection (e.g., lipopolysaccharide and *L.monocytogenes*^26^), partly through downregulation of autophagy^26^ and upregulation of mitochondrial injury^23^ and nuclear catastrophe.^22^ Knockin of HMGB1 in the heart protects mice against myocardial infarction.^25^ In contrast, a recent study from Robert Schwabe’s lab indicates that HMGB1 is not required for mitochondrial function and autophagy in the liver. In this study, the authors crossed HMGB1 conditional liver knockout mice with GFP-LC3 mice and then starved these mice for 24 h (n = 3 mice per group). The expression patterns of GFP-LC3 puncta and GFP-LC3 cleavage were similar between these mice upon starvation, suggesting that an HMGB1-independent autophagy system exists in the liver.^24^ Although the exact mechanism of this phenotype is not clear, a major difference between Robert Schwabe’s engineered HMGB1 mice and other groups is the tissue-level expression of HMGB1 after knockout. Mice with hepatocyte-specific deletion of *Hmgb1* from Robert Schwabe’s lab are not complete conditional knockout mice; the protein level of HMGB1 in the liver is decreased by about 70%.^24^ Thus, autophagy appears to correlate with HMGB1 protein level, and low HMGB1 levels may still sustain autophagy pathway activation. Moreover, the original GFP-LC3 mice study by Mizushima et al. demonstrated that the regulation of autophagy is tissue/organ-dependent and not restricted to a starvation response at 24 or 48 h.^27^

## Conclusions

It has become clear that HMGB1-dependent autophagy promotes chemotherapy resistance,^11^^,^^12^^,^^28^^-^^35^ sustains the tumor metabolism requirement^19^^,^^20^ and T cell survival,^36^ prevents polyglutamine aggregates^37^ and excitotoxicity,^38^ and protects against endotoxemia, bacterial infection, and ischemia-reperfusion injury.^26^^,^^39^^-^^41^ However, many questions remain unanswered regarding HMGB1-independent autophagy in the liver, including its tissue-specific role. HMGB1 dysfunction has been implicated in various forms of liver disease ranging from liver damage to fibrosis, as well as tumorigenesis.^42^ Extensive research is needed to determine the relationship between HMGB1, autophagy, and liver diseases. Of note, primary cells and cell lines have different baseline levels of autophagy as well as HMGB1 because transformed cell lines display different gene expression profiles.^43^ Understanding HMGB1-dependent and -independent autophagy in more detail will provide insight into the integrated stress response and guide HMGB1-based therapeutic intervention in cancer and other diseases.^44^

## Abbreviations:

ATG: autophagy-related
AGER/RAGE: advanced glycosylation end product-specific receptor
HMGB1: high mobility group box 1
HSPB1: heat shock 27 kDa protein 1
MAPK: mitogen-activated protein kinase
NACC1: nucleus accumbens associated 1, BEN and BTB (POZ) domain containing
ULK1: unc-51 like autophagy activating kinase 1
